# “What Did You Learn?” - An Alternative Narrative Approach to Student Evaluations of Teaching

**DOI:** 10.1177/23821205251332816

**Published:** 2025-05-04

**Authors:** Gunnar Tschudi Bondevik, Eivind Alexander Valestrand, Monika Kvernenes

**Affiliations:** 1 Department of Global Public Health and Primary Care, University of Bergen & NORCE Norwegian Research Centre, Bergen, Norway; 2 Center for Medical Education, Faculty of Medicine, 1658 University of Bergen, Bergen, Norway; 3 Center for Medical Education & Department of Clinical Medicine, Faculty of Medicine, 1658 University of Bergen, Bergen, Norway

**Keywords:** learning outcomes, medical education, student evaluation, student narratives, teaching quality

## Abstract

**Objective:**

In medical education, student evaluations of teaching (SETs) are commonly used as part of the quality assurance system. There are, however, concerns about the usefulness of traditional questionnaire-based SETs, as they have been found to correlate with factors unrelated to teaching quality. This article explores potential benefits of using an alternative method, shifting the students’ focus from evaluating the teaching to examining perceived learning outcomes.

**Methods:**

In 2023, we invited third and sixth year medical students at the University of Bergen, Norway, to write a reflection on their learning outcomes after completing a four days communication course and a two days consultation course, respectively. The 179 narratives were analysed qualitatively with a focus on what students chose to highlight, and how their reflections shed light on the quality of teaching. We also invited four teachers to read the students’ texts and report back on the usefulness of this approach to SETs.

**Results:**

Based on systematic text condensation we found that student narratives provided insights into learning activities, learning environment, learning outcomes and learning to be a doctor. The teachers advocated that producing the narratives might be beneficial for the students’ learning. They also valued the change in focus from teaching to learning, and the comprehensive information this approach to SET provided.

**Conclusions:**

Our findings suggest that, although the narrative approach to SETs is time-consuming, it provides the teachers with insight into the effectiveness of their teaching. Moreover, asking students to reflect on their learning outcomes may also benefit students.

## Introduction

In medical education, student evaluations of teaching (SETs) are frequently used as part of the quality assurance system. Although this practice empowers students, traditional questionnaire-based SETs have severe shortcomings that give cause to reconsider both the methods by which we collect student feedback and how we use their input to improve teaching. A number of factors give cause for concern about the usefulness of SETs: Student ratings often have nonresponse bias due to low response rates and have been shown to be biased favoring attractive and entertaining teachers.^1–3^ Students’ ability to recognize and evaluate quality in teaching can be questioned, which threatens the construct validity of the student feedback.^4–6^ Furthermore, there is poor evidence to claim that high-rated teachers are associated with better student learning outcomes.^7,8^ Finally, the way SETs are interpreted and followed up by teachers, program leaders, and deans have been criticized for putting too much weight on students’ opinions, for having unreasonable ramifications for teachers’ careers, and for not being used at all which challenges the ethics of collecting the feedback entirely.^9^ A recent review unequivocally concludes: “*In short, SET do not measure faculty's teaching effectiveness and their use in high-stakes personnel decisions is improper, unethical, and ought to be discontinued immediately”*.^10^

Various approaches have been suggested to rectify these adversities. One is challenging the methods used to collect feedback about teaching. As traditional questionnaire-based numeric teaching ratings rely on students responding to an end-of-course evaluation form, they represent a one-way type of communication that does little to encourage dialogue about what works, why and under what circumstances. Collecting student feedback by means of interviews, focus groups or student panels can be done during courses and allows adjustments to be done consecutively.^11–14^ Qualitative approaches have been shown to draw attention to a wider range of factors relevant to the perceived quality of teaching including the course content, the instructors and contextual factors such as scheduling issues and student composition.^15^ Programmatic evaluation of faculty effectiveness has been suggested, triangulating various methods for collecting student feedback and various sources to information about the quality of teaching to secure a more robust impression.^4,16^ Suggested stakeholders include administrative staff, the teachers themselves and students. Furthermore, faculty development initiatives for teachers with the intent to help them apply more suitable methods of collecting students’ feedback, how to interpret and apply them for improvement purposes, have been proposed.^17^ Although this might be considered a step towards more deliberate SET practices, it can be argued that it also frames SETs as the individual teachers’ responsibility, as opposed to seeing teaching, and education at large, as a collective and shared endeavor. This triggers questions about the overall purpose of collecting SETs. Depending on whether the evaluations are intended for leaders and deans to control and monitor the educational quality, for directors to develop their programs and courses, or if the purpose is to provide feedback to individuals or groups of teachers to enhance their professional development, SETs should be approached differently.

Moreover, there is an ongoing discourse challenging how we conceptualize quality in teaching. The questions asked in SETs mirror a definition of quality, and typically invite students’ feedback on organizational factors, student workload, teaching methods, assignments, assessment, teacher characteristics, and the course's contribution to their learning.^18^ Few questionnaires, although combining numeric ratings and written comments, ask students to reflect on what they have learned. By directing students’ attention towards administration and individual teachers’ performance, we risk ignoring that learning outcomes are results of students’ engagement in multiple activities in addition to teaching. Learning happens in formal and informal settings, affords various resources, and relies heavily not only on what teachers deliver to students, but also on how students choose to engage with the knowledge and skills they are expected to acquire. Teaching and learning thus become co-created rather than delivered.^19,20^ On the other hand, if good teaching is reduced to teaching that produces learning results, students’ input on their teaching and learning experiences becomes redundant. Hence, we risk framing the complex nature of education to a reductionist marked-oriented phenomenon aimed at producing learning with students as consumers and teachers as suppliers.^21,22^

With these challenges and shortcomings in existing teaching evaluation practices as a backdrop, Roxå et al,^1^ have suggested reconceptualizing SETs shifting the focus from measuring teaching quality towards accentuating what teaching is essentially for: enhancing student learning. In the reported project, we wanted to explore the potential benefits of not only collecting students’ feedback using an alternative method, but also the consequences of shifting the focus from evaluating the teaching and teachers’ performances as such, to focusing on the perceived learning outcomes of a given course. The rationale for this shift is anchored in the critique of traditional SETs as summarized above and reviewed in more detail by others.^1,4,23^

Our aim with the present study was threefold: we wanted to explore, by inviting students to reflect on their own learning outcomes, what they chose to highlight, how their reflections shed light on the quality of teaching and the teachers’ opinions about such an approach to SETs.

## Materials and Methods

### Study Design

We used a qualitative research design distributing a questionnaire containing one singular open-ended question: “What did you learn in the communication/consultation course?”. Responses were analysed through systematic text condensation, which is a method designed for thematic analysis of qualitative data.^24^

### Research Context

At the University of Bergen, Norway, third year medical students take a four-day clinical communication course, and sixth year students a two-day consultation course. Since these two courses deal with knowledge and skills that form the basis for patient communication across subjects in the medical curriculum, they were selected as suitable for our study. The purpose of the communication and consultation courses is to provide students with an understanding of the importance of the doctor-patient relationship, and to stimulate a lifelong development of empathy and patient-centered care. The courses seek to develop students’ competence in applying communication and clinical reasoning skills, technical skills, as well as reflect on the role of emotions and values embedded in daily clinical practice for the benefit of the patients. Students are trained to recognize good and poor communication and how to adapt patient-centered principles in clinical work.

Being an introduction to the topic, the communication course for third year medical students covers basic skills, while the consultation course for final year students is more advanced, as they have more clinical knowledge. Both courses, however, have a similar pedagogical approach with a combination of interactive plenary lectures, introducing and reinforcing theoretical patient-centered communication models and concepts, and group sessions with student active teaching methods such as roleplay with peer-to-peer feedback. In groups of 4–8 and guided by one of the teachers, students practice skills important for achieving good communication and they become aware of the impact of the way they communicate. Through group and plenary discussions, students are stimulated to critically reflect on what is considered good communication skills. By watching clinical videos, students are challenged to further discuss communication topics that are particularly difficult.

The 15 teachers involved in the third year communication course represent a number of clinical specialities, including academic specialists in general practice, internal medicine, surgery, anesthesiology, pediatrics, pulmonology and cardiology. The sixth year consultation course is run exclusively by 10 academic general practitioners. Communication and consultations skills are among the competences that are assessed during the Objective Structured Clinical Examinations (OSCE) at the end of the third and sixth years in medical school, a few months after completing the courses. Traditional SETs of previous communication and consultation courses have consistently achieved high ratings over several years, however, there has been uncertainty about the actual learning outcomes for students. This prompted an investigation into an alternative evaluation approach with a stronger emphasis on student learning.

### Data Collection

In January and February 2023, 102 third and 81 sixth year medical students at the University of Bergen were invited to participate in the present study. At the end of the final teaching session in the communication and consultation courses, the students were asked to write and anonymously submit a ½ page text of their learning outputs, prompted by the open-ended question “What did you learn in the communication/consultation course?”. As we wanted to study what and how the students addressed this single question, we chose not to provide more detailed instructions. Nor did we explicitly refer to the formal learning outcomes of the courses, which were available on the course websites. The summaries were not part of the students’ assessment.

Since it was incorporated as a learning activity in the teaching session, all attending students wrote a text. However, agreeing to have the text included as data in the research project was based on informed voluntary consent. As the study did not include health related information and was not affected by the Norwegian Health Research Act, approval from an ethics committee was not required. Following protocol for educational research, the project was registered in RETTE (project ID: R2724/2023), which is the University of Bergen's system for ensuring personal data are managed in accordance with the General Data Protection Regulation (GDPR) article 30. The reporting of this study conforms to the Standards for Reporting Qualitative Research (SRQR) (Supplementary file 1).

Two out of three course leaders for the communication course (the third being one of the authors) and both course leaders for the consultation course, who were also teaching in the courses, were given access to the corresponding anonymous student summaries a few days after the courses. These four teachers were purposefully sampled out of the total 25 communication and consultation teachers, because they were also course leaders being responsible for the overall quality of the courses. They were asked to submit a one-page reflection on the evaluation approach within one week, by answering the open-ended question “What kind of information did you get as a teacher by asking the students to write about what they had learned?”. Both student and teacher participation in the project was based on voluntary and informed consent.

### Data Analyses

The material was analyzed qualitatively by the authors of this paper, all being educators of medical students at the University of Bergen. Two of the authors are academic teachers and medical doctors, while the third is an academic teacher and medical education researcher with a background in higher education and university pedagogy. The analyses were done by using systematic text condensation - an established approach to analysing qualitative data influenced by phenomenological thematic analysis. It consists of the following four steps: 1) getting a general impression of the data, 2) identifying and sorting meaning units, 3) condensing the data to extract meaning, and 4) synthesising the condensed data.^24^ To get a general impression of the data and identify preliminary themes (step 1) the researchers must set aside their preconceptions and read the data with an open mind, focusing on the respondents’ voices. Next, the researchers read systematically through the transcripts and identify text fragments that are relevant for the study questions. These meaning units are sorted into code groups related to the themes. In step 3, contents from each code group are condensed or summarized into meaningful units. This entails abstracting overlapping statements from the data to represent patterns found in the data. Finally, the condensed data are synthesised as the researchers develop descriptions and concepts, thus providing insight into the research questions. It should be validated whether the synthesis is in accordance with the original context in the complete transcript.^24^

Initially, all authors reviewed the complete set of student narratives, in total 80 pages. Subsequently, each author independently identified various themes describing the learning experiences reported by the students. These preliminary themes were then discussed within the research group which identified meaning units related to the learning outcomes students emphasized and insights into the quality of teaching. Through this process, we developed code groups with subgroups covering diverse aspects of the students’ reported learning experiences. Following this, the first author synthesized the condensates into a preliminary set of descriptions, which were refined and finalised after extensive discussions and contributions from all authors. A similar approach was applied when analysing the four page material of feedback from the course leaders. Here, we searched for meaning units and devoloped code groups describing the teachers’ perspectives on this alternative evaluation method. Finally, we synthesized the condensates describing what the teachers gained from this evaluation approach, and their views on its strengths and limitations.

## Results

### The Students

All 81 sixth year medical students, 98 (96%) of the invited third year medical students, and all 4 invited teachers agreed to participate in the study. Based on our analyses we identified four main themes by which students’ reflections on learning outcomes provided insights about the quality of teaching: learning activities, learning environment, learning outcomes and learning to be a doctor. The teachers chose to highlight the change in focus from teaching to learning and a change to more comprehensive information with this approach of SETs.

### Learning Activities

The students described the organization of the courses and the teaching methods used. They discussed the course curriculum, its structure, and commented on the schedule and the teacher performances. Several students highlighted the value of pedagogical diversity, including plenary and group sessions, role plays, lectures, patient visits and videos. They appreciated the high degree of student engagement, especially in the group sessions. Despite initial discomfort and apprehension, students found group exercises with role plays to be very useful. Through repeated exercises they experienced improved learning outcomes.

Generally, I find role-playing to be an uncomfortable learning method, but I believe it can be useful for specific types of learning. Even though I found it intimidating, I definitely think there was a valuable learning outcome.’ (sixth year student no 32, Consultation course)

The students commented on the good learning outcomes associated with patients participating in the course, as they shared experiences with both good and poor communication with doctors. They also added that group sessions provided ample time for thorough discussions and reflections on various communication aspects. Students underscored the significance of receiving feedback both from their peers and teachers in the group exercises. They expressed that both providing feedback to others and receiving constructive criticism increased their awareness, and served as a motivational factor to improve their communication skills. Students reported that they had received instructions on various methods of delivering feedback, and asserted that this was beneficial for both theoretical studies and practical clinical situations. Some students wanted more feedback from the teachers, in addition to comments from their peers.

‘*It has been very nice to receive feedback from both fellow students and the group teacher. I believe we have learned useful methods for providing good and constructive feedback.’ (sixth year student no 38, Consultation course)*

### Learning Environment

A number of students chose to mention that establishing a learning environment characterised by a high degree of psychological safety was essential for achieving an optimal learning outcome. They acknowledged the liberating aspect of being permitted to not always know what to say or what to do in a patient consultation. This reflected back on both the student role (being allowed to not know because you are a learner) and the physician role (legitimizing uncertainty in patient communication). They appreciated the opportunities to adjust their communication habits in the group sessions, thereby enhancing their communications skills as well as their uncertainty tolerance.

‘*However, this improved over time ….. by becoming more comfortable with feeling a bit uncertain in conversations without it being the end of the world.’ (sixth year student no 75, Consultation course)*

Several students found that they developed close relationships with both peers and teachers during the courses. This was especially evident in the group sessions, where the limited number of students allowed them to get to know each other quite well throughout the course. The students noted that a safe group setting, combined with having the same group teacher throughout the course, facilitated the formation of these personal relations. They also believed that having teachers who prioritize building relationships is particularly crucial in this kind of courses involving challenging exercises about potentially vulnerable topics.

‘*Role-playing can quickly become an artificial and uncomfortable situation, but I believe that our group leader created favorable conditions that made this much more enjoyable than expected.’ (third year student no 5, Communication course)*

### Learning Outcomes

The students elaborated on learning outcomes from the courses, in particular the improvement of their communication and consultation skills and the importance of empathy and to explore the patient perspective. The latter was especially experienced in the group sessions.

‘*The course has provided me with a better foundation to deliver quality healthcare in a professional yet empathetic manner.’ (third year student no 38, Communication course)*

The students valued the opportunity to practice communications skills on sensitive topics such as sexual health, cancer and death. Additionally, they expressed how the acquired knowledge from the courses was seen as relevant to and interconnecting with other subjects in the curriculum. The students discussed the concept of “communication” and its essence, thus acknowledging that good communication and helping patients may be challenging. They described an increased understanding of the importance of communication skills, and appreciated the emphasis on person- and patient-centred approaches in the courses. Moreover, students asserted having a raised awareness about the impact of non-verbal communication and using body language. They stated that knowledge about communication strategies was perceived particularly relevant for the clinical setting.

‘*We have practiced using body language, pauses, and other techniques to make the patient comfortable and confident in us as healthcare providers.’ (third year student no 68, Communication course)*

Some students drew connections between how good communication skills can enhance the quality of consultations. They described strategies for achieving efficient consultations, using the various phases in a consultation model designed to structure medical interviews. The significance of initiating consultations with open-ended questions was underscored, as well as the impact of deliberate periods with silence during patient interactions. The students reported that the course had equipped them with an expanded repertoire for conducting consultations, including skills in motivational interviewing techniques.

‘*The consultation course taught me how to structure a consultation in order to elicit what is important for the patient.’ (sixth year student no 6, Consultation course)*

### Learning to be a Doctor

The students elaborated on how they saw the course contributing to shaping their professional identities. While recognising that they still had a lot to learn, they expressed a high motivation for further practice – emphasising that communication is a skill that can be developed. Some students described the importance of curiosity about patients and providing emotional support, stating that effective communication skills can significantly influence patient outcomes. They expressed a strong desire to become doctors proficient in communication.

‘*In the communication course, I’ve learned a lot about how to use the role of a doctor in a way that promotes safety and shows interest through communication.’ (third year student no 21, Communication course)*

The students particularly appreciated that group teachers shared their experiences as practicing doctors, giving them a glimpse into “real doctor work”. Participants highlighted their current roles as students and reflected on how group exercises had provided them with self-insight. They noted a growing awareness of their personal strengths and resources, and commented on how the combination of communication skills and clinical knowledge could improve relationships and trust in patient interactions. Despite being in a transitional phase between novice and experienced, both third year and sixth year students defined themselves as health professionals.

‘*I am left with a feeling of pride about how we as health professionals get to meet people at their most vulnerable.’ (third year student no 55, Communication course)*

In particular, sixth year students reflected on their future roles as medical doctors, bringing forward characteristics they deemed essential for being a good doctor in various clinical situations. They elaborated on the state of transition between being a student and a doctor, discussing how to maintain good and trusting doctor-patient relationships. They acknowledged feeling anxious about making mistakes in future clinical work, and the challenges of living with uncertainty.

‘*I have gained a much greater understanding of the approach during a consultation to establish a good relationship with the patient that opens up a conversation where all the issues the patient wants to discuss are brought forward, and, most importantly, makes the patient feel heard.’ (sixth year student no 7, Consultation course)*

### The Teachers/Course Leaders

The analyses of the teachers’ comments to the student texts revealed changes along two axes: One relates to how the evaluation method entailed a horizontal change in perspective from focusing less on teacher performance and more on students’ learning. The other axis represents a vertical change as the texts provided the teachers with richer and more in-depth insight into how the course was perceived by students. Both axes are elaborated below.

### Changed Focus From Teaching to Learning

The teachers characterized this alternative method as a shift from a teacher-centred to a student-centred evaluation. They noted that students may choose what they want to emphasize in the evaluation. Hence the students communicate what they consider important in their learning. One teacher summarized that this evaluation method benefits the students, allowing them the opportunity to reflect on their own learning. Additionally, a teacher suggested that the act of writing the student text is likely to reinforce key messages in the teaching, emphasising that students in this manner take greater responsibility for their own learning.

‘*Changing the focus to what the students themselves perceive they have gained from the course gives us a completely different perspective. It shifts the focus from assessing the lecturer's “performance” to the students’ own learning outcomes.’ (Teacher no 3)*

One teacher expressed a sense of relief, noting that the focus shifted away from spotlighting teaching performance to emphasising what the students had learned.

‘*I usually dread evaluations that are structured with praise/criticism, what was good? What could have been better?…… But the question “what have you learned…?” leads to students answering in a way that is less intimidating for me.’ (Teacher no 2)*

### Change Towards More Comprehensive Information

The teachers explained how the student texts, offering more in-depth and nuanced information, were helpful for developing the courses further, stating that they gained more insight from this type of evaluation than from traditional SETs. For example, one teacher claimed that students appeared to be more honest in this type of evaluation, providing more specific feedback. Another teacher added that the narratives gave greater insight into the students’ understanding.

‘*I believe that this type of feedback stimulates greater reflection on the topics that have been taught, rather than a fact-based presentation of the learning outcomes.’ (Teacher no 4)*

The teachers also raised some concerns regarding this evaluation method. One teacher commented on the substantial volume of students’ narratives (40-50 pages per course), which was perceived as demanding to read. Although teachers indicated that this type of SET appeared to be more reliable than traditional evaluations, one teacher expressed concern about potential “eager to please” attitude in the student texts. Another teacher recommended supplementing this alternative approach to SET with an oral evaluation of the courses.

‘*I think we can rely more on this type of evaluation than a “good” evaluation in the “old-fashioned” way. However, there is still a LOT of text to deal with when there are so many students.’ (Teacher no 1)*

## Discussion

This study set out to explore how students’ reflections on learning outcomes could provide insights about the quality of teaching and thus provide an alternative to traditional SETs. Our results indicate that the alternative approach representing a shift from focusing on teaching performances to focusing on learning, provided feedback that helped teachers evaluate their teaching. As students reflected upon their learning outcomes, they also provided insights into how they perceived the learning activities, learning environment, and how they perceived the course content relevant for future clinical practice. As such, the themes identified in students’ writing provided insight into how students understand quality in education, without asking them to define what is good teaching. The themes all identified factors considered essential for learning. Furthermore, they were seen as interconnected in the sense that the learning environment and learning activities impact students’ learning outcomes. Consequently, they are also part of forming their emerging professional identities, as “the process of coming to know has person-forming qualities”.^25^

Overall, the learning outputs described by students aligned well with the pre-defined learning outcomes for the courses. Additionally, this evaluative method gave insights into the students’ reflections on their future professional work. As a central goal of medical education is to help students develop how to “think, act, and feel like a physician”, evaluative methods that provide educators with insights into students’ identity formation are necessary to be able to help students develop as future professionals.^26,27^ Traditional SETs focusing on the teachers performance do not invite students’ self-reflection nor let teachers access those reflections. The evaluation method presented in this study enables a desired integration suggested by Biesta and van Braak^28^ of information about how students reflect on their knowledge acquisition, how they feel integrated into the social group of doctors, and their personal journey of forming values and becoming a professional.

The teachers in our study expressed relief that this way of evaluating took some of the pressure off their performance and instead highlighted what and how the students learned. This created space for teachers to integrate their own teaching experience with students’ input and thus draw contextualized conclusions about the quality of teaching and areas for further improvement. This relates to Biggs proposed highest level of teaching competence, in which educators teaching for enhanced learning need to pay attention to “what the student does”.^29^

This method of teaching evaluation explicitly places the responsibility on the educators to make sense of and extract the essential points from the feedback on a teaching or learning activity, a course, or a curriculum. The evaluation provides educators with insights into what students are aware of having learned, and the educator then needs to consider how the students’ reflections on their learning play into the teaching context. It may be part of this method's strength that it forces educators to use their pedagogical competence to make student feedback helpful in developing their teaching. Other evaluation methods, like students’ ratings of teachers, may have an attractive simplicity that can make them improperly used if one (often wrongly) believes they have good validity and are not confounded by irrelevant factors.^4^

Our findings give support for outlining several benefits of a learning-centered narrative evaluation approach: Firstly, students’ learning can be enhanced by having them reflect upon the course content and perceived learning outcomes. Secondly, students’ awareness of how their learning is not only a matter of teachers delivering the right content in the right manner, but also a product of their own investments and engagement in the course. Thirdly, teachers and course organizers may benefit from insight into what the teaching has achieved as an onset for improving the course for future students. The mix of explicit and implicit feedback embedded in the students’ reflections on learning outcomes provides more in-depth information and constructive suggestions compared to numeric SET scales that merely pass judgment. Making clear that the writing process has personal benefits for students may motivate them more to take part in the evaluation than appealing to their solidarity with future students. Finally, our experiment suggests that teachers benefit from feeling less scrutinized which relieves some of the destructive performative pressure associated with teaching.

We also identified constraints to this evaluation method: The variety of information the students’ feedback can hold may invite teachers to biasedly focus on what they are most interested in. Essential feedback may be overlooked, neglected, or challenging to locate in a plethora of feedback. Furthermore, the approach is time-consuming, especially in large student groups. Limiting the length of students’ text and/or reading a random selection of texts may remedy this. Depending on the heterogeneity and depth of the feedback, and the purpose of analysis, saturation is likely to be achieved by reading 20–30% of the responses in large classes. As students often misjudge their own learning,^30^ teachers should recognize that students’ texts primarily represent their reflections and immediate awareness of learning. Other methods need to be applied to assess the actual learning of cognitive, affective, and psychomotor skills. Furthermore, we recommend using this evaluation method when there is a need for deep exploration of students’ learning experiences in a course. The time used may be well worth it if it gives access to information that enables changes that can enhance students’ learning. Hujala et al,^31^ have developed a process for analysing large amounts of written SETs, connecting qualitative findings to theories and quantitative feedback. Artificial intelligence tools like ChatGPT might also be useful for educators to summarize feedback from a large number of students. However, we believe that this should be accompanied by reading at least a selection of the original SETs, as important information may otherwise be lost.

### Implications

The courses that formed the base for our study incorporated diverse teaching methods and learning activities, leading us to confidently recommend this evaluative approach across various types of instruction, both theoretical and practical, and at different stages of a medical program. However, there is not, and should not be, one universal evaluation method applicable for all purposes. Instead, we offer this learning-centered approach as a suggestion for how to mitigate some of the challenges associated with traditional scale-based teacher-centered SETs. The choice of evaluation method needs to be adapted to the purpose of the evaluation. We urge educators to explore and expand their repertoire of evaluation methods to include data like students’ stories or reflections on what they have learned.

Moreover, we encourage all educators to discuss, reflect, and become aware of what they want to focus their evaluation on, not merely collect the data they can and use it without any foreseen direction or focus. The choice of evaluation method may partly be a question of an organization's evaluation culture, how efficiency or quality in teaching is understood, valued, and rewarded, and how the individual educator's mindset motivates them to invest time and effort in their teaching.

### Strengths and Limitations

There are some limitations to using systematic text condensation.^24^ During the four steps of analysis, some information will be lost, and the individual context may be less clear. Summaries of the participants’ texts may also lead to varying interpretations. However, in the final step of systematic text condensation, the researchers should ensure that the results reflect the initial full transcript. The steps of systematic text condensation are precise, allowing a systematic approach to the analyses. As with qualitative studies in general, reflexivity is a precondition for the quality of the research.

The communication/consultation courses in question have history of being well organized and well received by students for several years, and has a dedicated pool of teachers. We do not know how this evaluation approach would have been received in courses needing greater revision, or if the widely applicable course content affected our results. Hence, further research is needed to study the applicability of this evaluation approach in other teaching settings in the medical curriculum and in other fields of study.

## Conclusions

Traditional SETs have been found to correlate with factors unrelated to the quality of teaching and its efficiency in achieving the intended learning outcomes. Our study indicates that asking students to reflect on what they have learned, as opposed to inviting them to evaluate teaching performances, produces valid feedback which may benefit both students and teachers by offering detailed insights into what is achieved by teaching.

## References

[1] Roxå T Ahmad A Barrington J Van Maaren J Cassidy R . Reconceptualizing student ratings of teaching to support quality discourse on student learning: a systems perspective. High Educ. 2022;83(1):35-355. doi: 10.1007/s10734-020-00615-1

[2] Morrison K . Online and paper evaluations of courses: a literature review and case study. Educ Res Eval. 2013;19(7):585-604. doi: 10.1080/13803611.2013.834608

[3] Goos M Salomons A . Measuring teaching quality in higher education: assessing selection bias in course evaluations. Res High Educ. 2017;58(4):341-364. doi: 10.1007/s11162-016-9429-8

[4] Ginsburg S Stroud L . Necessary but insufficient and possibly counterproductive: the complex problem of teaching evaluations. Acad Med. 2023;98(3):300-303. doi: 10.1097/ACM.0000000000005006 36538693

[5] Deslauriers L McCarty LS Miller K Callaghan K Kestin G . Measuring actual learning versus feeling of learning in response to being actively engaged in the classroom. Proc Natl Acad Sci USA. 2019;116(39):19251-19257. doi: 10.1073/pnas.1821936116 31484770 PMC6765278

[6] Hessler M Pöpping DM Hollstein H , et al. Availability of cookies during an academic course session affects evaluation of teaching. Med Educ. 2018;52(10):1064-1072. doi: 10.1111/medu.13627 29956364

[7] Uttl B White CA Gonzalez DW . Meta-analysis of faculty’s teaching effectiveness: student evaluation of teaching ratings and student learning are not related. Stud Educ Eval. 2017;54(1):22-42. doi: 10.1016/j.stueduc.2016.08.007

[8] Clayson DE . Student evaluations of teaching: are they related to what students learn? A meta-analysis and review of the literature. J Market Educ. 2009;31(1):16-30. doi: 10.1177/0273475308324086

[9] Borch I Sandvoll R Risør T . Discrepancies in purposes of student course evaluations: what does it mean to be “satisfied”? Educ Assess Eval Account. 2020;32(1):83-102. doi: 10.1007/s11092-020-09315-x

[10] Uttl B . Lessons learned from research on student evaluation of teaching in higher education. In: Rollett W Bijlsma H Röhl S , eds. Student Feedback on Teaching in Schools: Using Student Perceptions for the Development of Teaching and Teachers. Springer; 2021:237-256. doi: 10.1007/978-3-030-75150-0_15

[11] Cathcart A Greer D Neale L . Learner-focused evaluation cycles: facilitating learning using feedforward, concurrent and feedback evaluation. Assess Eval Higher Educ. 2014;39(7):790-802. doi: 10.1080/02602938.2013.870969

[12] Berk RA . Survey of 12 strategies to measure teaching effectiveness. Int J Teach Learn Higher Educ. 2005;17(1):48-62.

[13] Steyn C Davies C Sambo A . Eliciting student feedback for course development: the application of a qualitative course evaluation tool among business research students. Assess Eval Higher Educ. 2019;44(1):11-24. doi: 10.1080/02602938.2018.1466266

[14] Brandl K Rabadia SV Chang A Mandel J . Benefits of focus group discussions beyond online surveys in course evaluations by medical students in the United States: a qualitative study. J Educ Eval Health Prof. 2018;15(1):1-5. doi: 10.3352/jeehp.2018.15.25 PMC6249141 30321914

[15] Alhija FNA Fresko B . Student evaluation of instruction: what can be learned from students’ written comments? Stud Educ Eval. 2009;35(1):37-44. doi: 10.1016/j.stueduc.2009.01.002

[16] Jahangiri L Mucciolo TW Choi M Spielman AI . Assessment of teaching effectiveness in US dental schools and the value of triangulation. J Dent Educ. 2008;72(6):707-718. doi: 10.1002/j.0022-0337.2008.72.6.tb04536.x 18519601

[17] Spooren P . Improving the quality of teaching. *Assessing and Enhancing Student. Experience in Higher Education*. 2021:159–190. doi: 10.1007/978-3-030-80889-1_7

[18] Bush MA Rushton S Conklin JL Oermann MH . Considerations for developing a student evaluation of teaching form. Teach Learn Nurs. 2018;13(2):125-128. doi: 10.1016/j.teln.2017.10.002

[19] Bovill C Cook-Sather A Felten P Millard L Moore-Cherry N . Addressing potential challenges in co-creating learning and teaching: overcoming resistance, navigating institutional norms and ensuring inclusivity in student–staff partnerships. High Educ. 2016;71(2):195-208. doi: 10.1007/s10734-015-9896-4

[20] Könings KD Mordang S Smeenk F Stassen L Ramani S . Learner involvement in the co-creation of teaching and learning: AMEE Guide No. 138. Med Teach. 2021;43(8):924-936. doi: 10.1080/0142159X.2020.1838464 33153367

[21] Biesta G . Good education in an age of measurement: on the need to reconnect with the question of purpose in education. Educ Assess Eval Account. 2009;21(1):33-46. doi: 10.1007/s11092-008-9064-9

[22] Magnússon G Rytzler J . Towards a Pedagogy of Higher Education: The Bologna Process, Didaktik and Teaching. Routledge; 2022. doi: 10.4324/9781003054160

[23] Constantinou C Wijnen-Meijer M . Student evaluations of teaching and the development of a comprehensive measure of teaching effectiveness for medical schools. BMC Med Educ. 2022;22(1):113. doi: 10.1186/s12909-022-03148-6 35183151 PMC8858452

[24] Malterud K . Systematic text condensation: a strategy for qualitative analysis. Scand J Public Health. 2012;40(8):795-805. doi:10.1177/1403494812465030 23221918

[25] Barnett R . Knowing and becoming in the higher education curriculum. Stud Higher Educ. 2009;34(4):429-440. doi: 10.1080/03075070902771978

[26] Merton RK Reader G Kendall PL . The Student-Physician: Introductory Studies in the Sociology of Medical Education. Harvard University Press; 1957. doi: 10.4159/arvard.9780674366831

[27] Cruess RL Cruess SR Boudreau JD Snell L Steinert Y . Reframing medical education to support professional identity formation. Acad Med. 2014;89(11):1446-1451. doi: 10.1097/ACM.0000000000000427 25054423

[28] Biesta GJJ van Braak M . Beyond the medical model: thinking differently about medical education and medical education research. Teach Learn Med. 2020;32(4):449-456. doi: 10.1080/10401334.2020.1798240 32799696

[29] Biggs J . What the student does: teaching for enhanced learning. High Educ Res Dev. 1999;18(1):57-75. doi: 10.1080/0729436990180105

[30] Blanch-Hartigan D . Medical students’ self-assessment of performance: results from three meta-analyses. Patient Educ Couns. 2011 Jul;84(1):3-9. Epub 2010 Aug 14. PMID: 20708898. doi: 10.1016/j.pec.2010.06.037 20708898

[31] Hujala M Knutas A Hynninen T Arminen H . Improving the quality of teaching by utilising written student feedback: a streamlined process. Comput Educ. 2020;157(1):103965. doi: 10.1016/j.compedu.2020.103965

